# The four and a half LIM domains 2 (FHL2) regulates ovarian granulosa cell tumor progression via controlling AKT1 transcription

**DOI:** 10.1038/cddis.2016.207

**Published:** 2016-07-14

**Authors:** G Hua, C He, X Lv, L Fan, C Wang, S W Remmenga, K J Rodabaugh, L Yang, S M Lele, P Yang, A R Karpf, J S Davis, C Wang

**Affiliations:** 1 Olson Center for Women's Health, Department of Obstetrics/Gynecology, University of Nebraska Medical Center, Omaha, NE 68198, USA; 2 Key Lab of Agricultural Animal Genetics, Breeding and Reproduction of Ministry of Education, College of Animal Science and Technology, Huazhong Agricultural University, Wuhan, Hubei province 430070, China; 3 Department of Pathology and Microbiology, University of Nebraska Medical Center, Omaha, NE 68198, USA; 4 Department of Obstetrics, Gynecology & Reproductive Sciences, University of Maryland School of Medicine, Baltimore, MD 21201, USA; 5 Fred & Pamela Buffett Cancer Center, University of Nebraska Medical Center, Omaha, NE 68198, USA; 6 Omaha Veterans Affairs Medical Center, Omaha, NE 68105, USA

## Abstract

The four and a half LIM domains 2 (FHL2) has been shown to play important roles in the regulation of cell proliferation, survival, adhesion, motility and signal transduction in a cell type and tissue-dependent manner. However, the function of FHL2 in ovarian physiology and pathology is unclear. The aim of this study was to determine the role and functional mechanism of FHL2 in the progression of ovarian granulosa cell tumors (GCTs). Immunohistochemical analysis indicated that FHL2 was overexpressed in GCT tissues. Cellular localization of FHL2 in GCT cells was cell cycle dependent. Knockdown of FHL2 suppressed GCT cell growth, reduced cell viability and inhibited cell migration. Consistently, ectopic expression of FHL2 in GCT cells with very low endogenous FHL2 promoted cell growth, improved cell viability and enhance cell migration. Importantly, overexpression of FHL2 promoted GCT progression *in vivo*. Mechanistic studies indicated that FHL2 regulates *AKT1* gene expression *in vitro* and *in vivo*. Knockdown of FHL2 or AKT1 in GCT cell lines induced very similar phenotypes. Ectopic expression of constitutively active AKT1 rescued FHL2 knockdown-induced arrest of GCT cell growth and reduction of GCT cell viability, suggesting that FHL2 regulates GCT cell growth and viability through controlling AKT1 expression. Finally, co-immunoprecipitation and chromatin immunoprecipitation analyses indicated that FHL2 functions as a co-activator of NF*κ*B and AP-1 to regulate *AKT1* gene transcription. In conclusion, results from the present study indicate that FHL2 exerts its oncogenic action in GCT cells via controlling *AKT1* gene expression. FHL2 is a promising target for the development of novel drugs against ovarian granulosa cell tumor.

Granulosa cell tumors (GCTs) of the ovary account for ~80% of ovarian sex-cord/stromal tumors and are the most poorly understood ovarian neoplasms.^1, 2^ Although GCTs grow relatively slow, these tumors are characterized by their high frequency of recurrence, malignant potential and metastatic capacity.^2^ Recurrence of GCTs is associated with a high mortality rate, with 70–80% of women with recurrent disease succumbing to their tumors.^3, 4^ Metastasis of these tumors has been reported and can involve any organ.^5^ The presence of extraovarian disease correlates with a 5-year survival of 33–50%.^6^ In addition, excessive estrogen production by these tumors stimulates the endometrium, leading to the development of endometrial hyperplasia in 30–50% of patients and endometrial adenocarcinoma in 8–33% of patients. Some patients also present with symptoms of androgen excess.^7^

The etiology of GCT is not clear and less studied. FOXL2 has been identified as a potential driver in the pathogenesis of adult-type GCTs.^8, 9, 10^ Our previous studies indicated that the Hippo/YAP pathway may play an important role in the regulation of GCT cell proliferation, migration and steroidogenesis.^11^ Despite this progress, the molecular mechanisms underlying GCT development are largely unknown.

The four and a half LIM domains 2 (FHL2) contains four and a half highly conserved cysteine-rich LIM homeodomains. This unique structure enables FHL2 to interact with many different proteins.^12^ It is reported that FHL2 serves as a transcriptional co-activator of several transcription factors, including androgen receptor, AP-1, CREB, BRCA1 and WT-1.^13, 14, 15, 16^ Interestingly, FHL2 is also able to function as a transcriptional co-repressors of ERK2, PLZF, Nur77, E4F1 and FOXO1.^17, 18, 19^ FHL2 is expressed in a wide range of organs and tissues and plays critical roles in their physiology and pathology.^20, 21, 22^

The role of FHL2 in cancer is particularly intriguing because it functions as an oncogenic protein or a tumor suppressor.^22^ FHL2 acts as an oncogene in breast cancer,^23^ gastric and colon cancer,^24, 25^ prostate cancer,^15, 19, 26^ and glioblastoma.^27^ On the contrary, FHL2 has also been identified as a tumor suppressor in human rhabdomyosarcoma,^20^ hepatocellular carcinoma,^28^ neuroblastoma^29^ and a sub-type of breast cancer.^30^ The exact mechanism underlying its differential actions in different type of cancers is unclear.

It has been reported that FHL2 is overexpressed in the epithelial ovarian cancer tissues and is involved in the formation of focal adhesions.^31^ However, its role and functional mechanism(s) in ovarian cancer development and progression have not been studied. A very recent study indicated that FHL2 is spatio-temporally expressed in the ovarian granulosa cells,^32^ suggesting that FHL2 may play an important role in regulation of granulosa cell function and ovarian follicle development. Nevertheless, the role of FHL2 in ovarian granulosa cell pathology is largely unknown.

In the present study, we demonstrate that FHL2 plays a critical role in the initiation and progression of GCT. We found that FHL2 was overexpressed in human GCT tumor tissues. Overexpression of FHL2 in GCT cells increased cell viability and promoted cell growth, while knockdown of FHL2 reduced cell viability and suppressed GCT proliferation. Intriguingly, our mechanistic studies indicate that AKT1 is a target of FHL2 in GCT cells. FHL2 controls GCT cell viability and growth via regulating *AKT1* gene transcription.

## Results

### FHL2 is overexpressed in human GCT tissues

**Figure 1:**
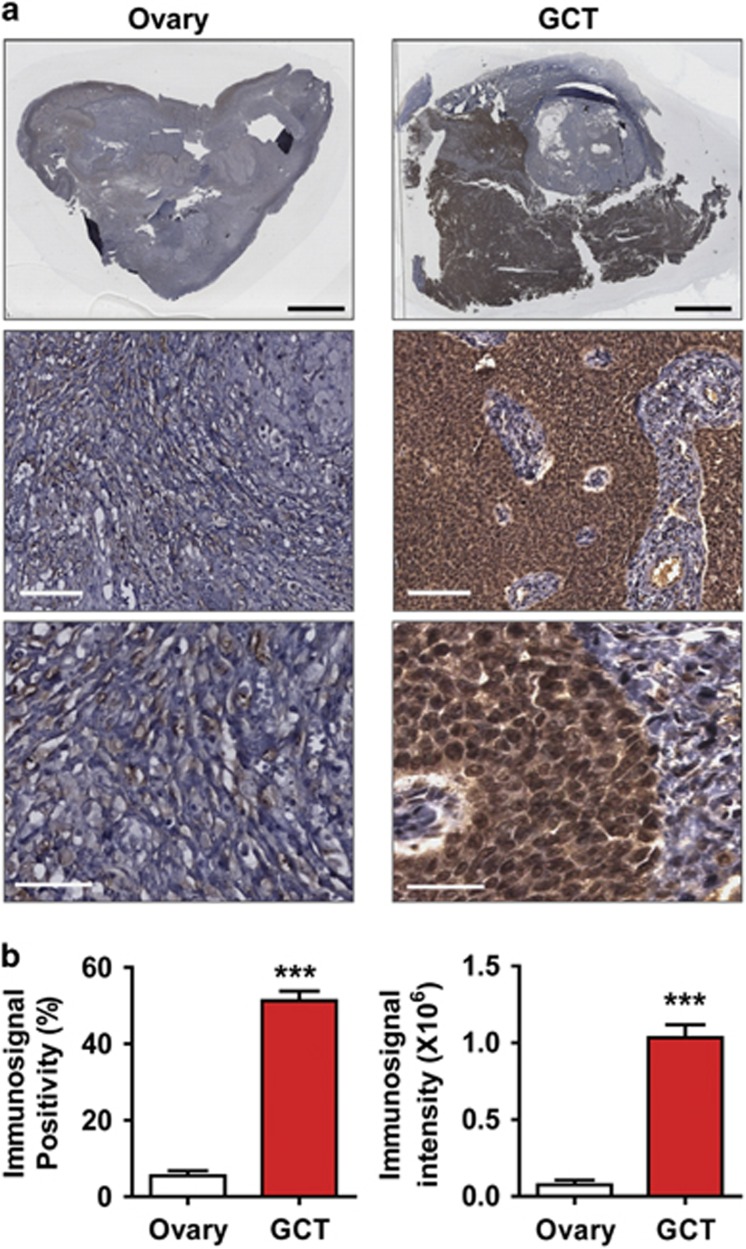
Expression of FHL2 in normal ovary tissues and GCT tumor tissues. (**a**) Representative images showing expression of FHL2 in normal ovarian tissues (left) and human GCT tumor tissues (right) detected by immunohistochemistry. FHL2 was stained in brown. Nuclei were counterstained with hematoxylin. Scale bar: 5 mm in the top panels, 100 *μ*m in the middle panels and 50 *μ*m in the lower panels. (**b**) Quantitative results showing the difference of positivity (percentage of the FHL2-positive cell number relative to the total cell number) and relative intensity of FHL2 immunosignal between the normal human ovarian tissues and GCT tumor tissues. Each bar represents mean±S.E.M. (*n*=10 for normal ovary; *n*=12 for GCT). ***P<0.001 compared with control

FHL2 expression was determined by immunohistochemistry in age-matched normal human ovarian tissues and GCT tumor tissues. The FHL2 protein level in the GCT tumor cells significantly increased compared with the age-matched normal control tissues (Figure 1a). Quantification of the FHL2 immunosignal indicated that both the immunosignal positivity (percentage of the FHL2-positive cells relative to the total cells) and immunosignal intensity in the GCT tumor tissues were significantly higher than in the control tissues (Figure 1b, *P*<0.001).

### FHL2 promotes GCT cell growth, viability and migration *in vitro*

**Figure 2:**
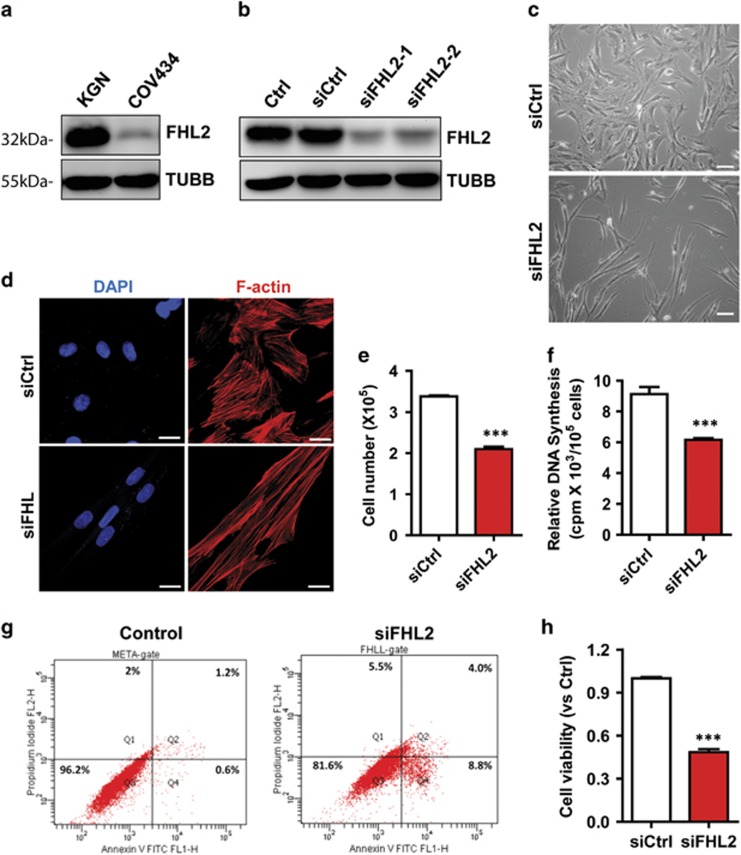
Knockdown of FHL2 in KGN cells suppressed cell growth and induced dramatic morphological change. (**a**) FHL2 protein levels in KGN and COV434 cells detected by western blot. (**b**) FHL2 protein levels in KGN cells transfected with non-targeting control siRNA (siCtrl) or FHL2 siRNAs (two different FHL2 siRNAs marked as siFHL2-1 and siFHL2-2). FHL2 levels were determined by western blot. *β*-Tubulin (TUBB) was used as a protein loading control. (**c**) Morphological change of KGN cells transfected with non-targeting control siRNA (siCtrl, upper panel) or FHL2 siRNA (siFHL2, lower panel). Scale bar: 25 *μ*m. (**d**) Representative images showing the actin cytoskeleton in KGN cells transfected with control siRNA (siCtrl, upper panel) or FHL2 siRNA (siFHL2, lower panel). Actin filaments (red) were stained with rhodamine-phalloidin. Nuclei were stained with DAPI (blue), Scale bar: 10 *μ*m. (**e**) Quantitative data showing cell growth change in KGN cells transfected with non-targeting control siRNA (siCtrl) or FHL2 siRNA (siFHL2). ***P<0.001 compared with control (siCtrl). (**f**) Knockdown of FHL2 significantly inhibited KGN cell DNA synthesis. DNA synthesis was determined by ^3^H-thymidine incorporation assay. Each bar represents mean±S.E.M. (*n*=5). ***P<0.001 compared with control (siCtrl). (**g**) Knockdown of FHL2 drastically promotes KGN cell apoptosis. Cells were stained with an Annexin V-FITC/PI dual staining kit and analyzed using the flow cytometry. Experiments were repeated for three times and the representative graphs were presented. Please note the drastic increase in apoptotic and dead cells (located in Q1, Q2 and Q4) in FHL2-knockdown KGN cells (siFHL2). (**h**) Quantitative data showing changes in the viability of KGN cells transfected with control siRNA (siCtrl) or FHL2 siRNA (siFHL2). ***P<0.001 compared with control (siCtrl)

The KGN cell line was derived from a recurrent ovarian granulosa cell carcinoma.^33^ The COV434 cell line was derived from a metastatic granulosa cell carcinoma.^34^ FHL2 was differentially expressed in KGN and COV434 cell lines. KGN cells expressed higher FHL2 protein, while COV434 cells expressed a very low amount of FHL2 protein (Figure 2a). FHL2 was mainly localized to the stress fibers in KGN cells in the interphase of the cell cycle (Supplementary Figure S1). However, in dividing KGN cells, the FHL2 protein level increased and the association between FHL2 and stress fibers reduced, leading to the even distribution of this protein in the cytoplasm. After cytokinesis, FHL2 protein set up their association with stress fibers again (Supplementary Figure S1).

To examine whether FHL2 plays a role in GCT tumor cell growth, we used FHL2 siRNA to knock down FHL2 in KGN cells. Western blot results indicated that both siRNAs efficiently reduced the FHL2 to ~30% of the original protein level (Figure 2b). We found that knockdown of FHL2 induced elongation of KGN cells, leading to slim and fibroblast-like cellular morphology (Figure 2c). Fluorescent microscopy showed that the actin filaments in FHL2-knockdown KGN cells elongated, clustered and formed many thick and long stress fiber bundles (Figure 2d). Knockdown of FHL2 also suppressed KGN cell growth (Figure 2e), reduced DNA synthesis (Figure 2f), arrested cell cycle (Table 1) and suppressed levels of pro-proliferation proteins such as CDT1, MCM2, MCM7, cyclin B1 and cyclin E (Supplementary Figure S2a). Moreover, knockdown of FHL2 blocked TGF*α*-induced cell cycle progression (Supplementary Figure S2b). In addition, Annexin V-FITC/PI-based flow cytometry and MTT assays indicated that knockdown of FHL2 in KGN cells significantly reduced cell viability (Figures 2g and h). Finally, wound-healing assays and the trans-well migration analyses clearly showed that knockdown of FHL2 also inhibited KGN cell migration (Supplementary Figures S3a and b).

**Figure 3:**
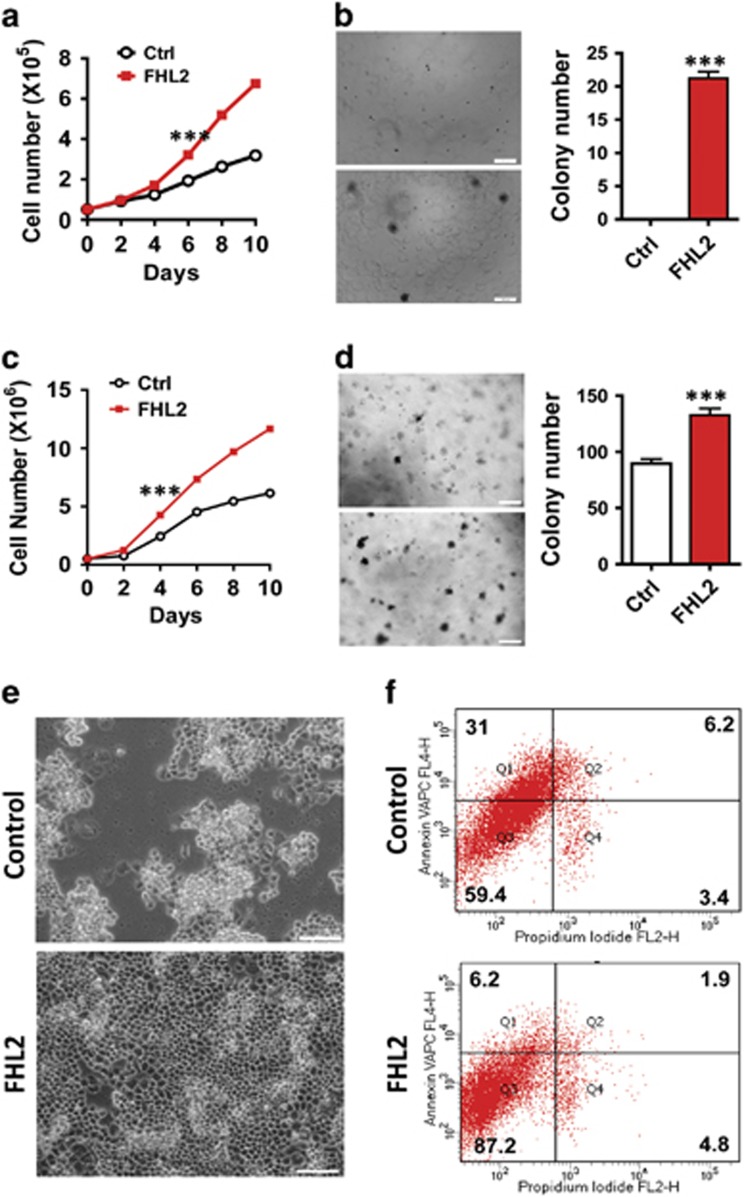
Ectopic expression of FHL2 enhances GCT cell proliferation and survival. (**a**) Growth curve of KGN cells transfected with control vector (Ctrl) or FHL2-expressing vectors (FHL2). Each point represents mean±S.E.M. of four independent experiments. ***P<0.001 compared with control (Ctrl) on day 6. (**b**) Left: representative images showing colony formation in KGN cells transfected with control vector (Ctrl) or FHL2-expressing vectors (FHL2). Scale bar: 200 *μ*m. Right: quantitative data showing the colony number formed in control and FHL2-overexpression KGN cells. ***P<0.001 compared with control (Ctrl). (**c**) Growth curve of COV434 cells transfected with control vector (Ctrl) or FHL2-expressing vectors (FHL2). Each point represents mean±S.E.M. of four independent experiments. ***P<0.001 compared with control (Ctrl) on day 4. (**d**) Left: representative images showing colony formation in COV434 cells transfected with control vector (Ctrl) or FHL2-expressing vectors (FHL2). Scale bar: 200 *μ*m. Right: quantitative data showing the colony number formed in control and FHL2-overexpression COV434 cells. ***P<0.001 compared with control (Ctrl). (**e**) Morphology of cultured COV434 cells transfected with empty control (control) vector or FHL2 expression vector (FHL2). Note the spread of the COV434 cells with ectopic expression of FHL2 (lower panel). (**f**) Changes in the viability of COV434 cells transfected with empty control vector (control) or FHL2-expressing vector (FHL2). Cells were stained with an Annexin V-APC/PI dual staining kit and analyzed using the flow cytometry. Experiments were repeated for three times and the representative graphs were presented

To further confirm the role of FHL2 in GCT cell viability and growth, we transfected KGN and COV434 cell lines with a lentivirus-based FHL2 expression vector or an empty control vector (control). Overexpression of FHL2 in KGN cells significantly increased the cell growth rate (Figure 3a), and promoted cell cycle progression (Supplementary Figure S4a). Although KGN cells are derived from a malignant GCT, they did not readily form colonies in a soft agar culture system. However, KGN cells transfected with the FHL2-expressing vector formed colonies in the soft agar culture system (Figure 3b and Supplementary Figure S4b), suggesting that FHL2 supports the anchorage-independent growth of KGN cells. Similarly, ectopic expression of FHL2 in COV434 cells significantly stimulated COV434 cell growth (Figure 3c) and greatly promoted colony formation of COV434 cells in soft agar culture system (Figure 3d and Supplementary Figure S4c, *P*<0.001).

Ectopic expression of FHL2 also greatly enhanced GCT cell survival. Starvation of KGN cells by depriving cells of FBS for 21 days induced cell apoptosis (Supplementary Figure S5a). However, ectopic expression of FHL2 prevented KGN cells from starvation-induced apoptosis, and successfully maintained KGN cell morphology (Supplementary Figure S5). COV434 cells are featured by their unique growth pattern. Once the first cohort of cells attach to the culture plate, their progeny grow on the top of the attached cells to form clusters (Figure 3e). Flow cytometry showed that COV434 cells have a very high rate of apoptosis, even in growth medium containing 10% FBS (Figure 3f). In contrast, FHL2-expressing COV434 cells spread out and form a monolayer in culture plate (Figure 3e). Flow cytometry showed that ectopic expression of FHL2 significantly reduced apoptosis rates and thereby supported COV434 cell growth (Figure 3f). In addition, wound-healing assays showed that the ectopic expression of FHL2 in KGN cells significantly promoted GCT cell mobility (Supplementary Figure S6).

### Elevated FHL2 promotes GCT cell growth, viability and migration *in vivo*

**Figure 4:**
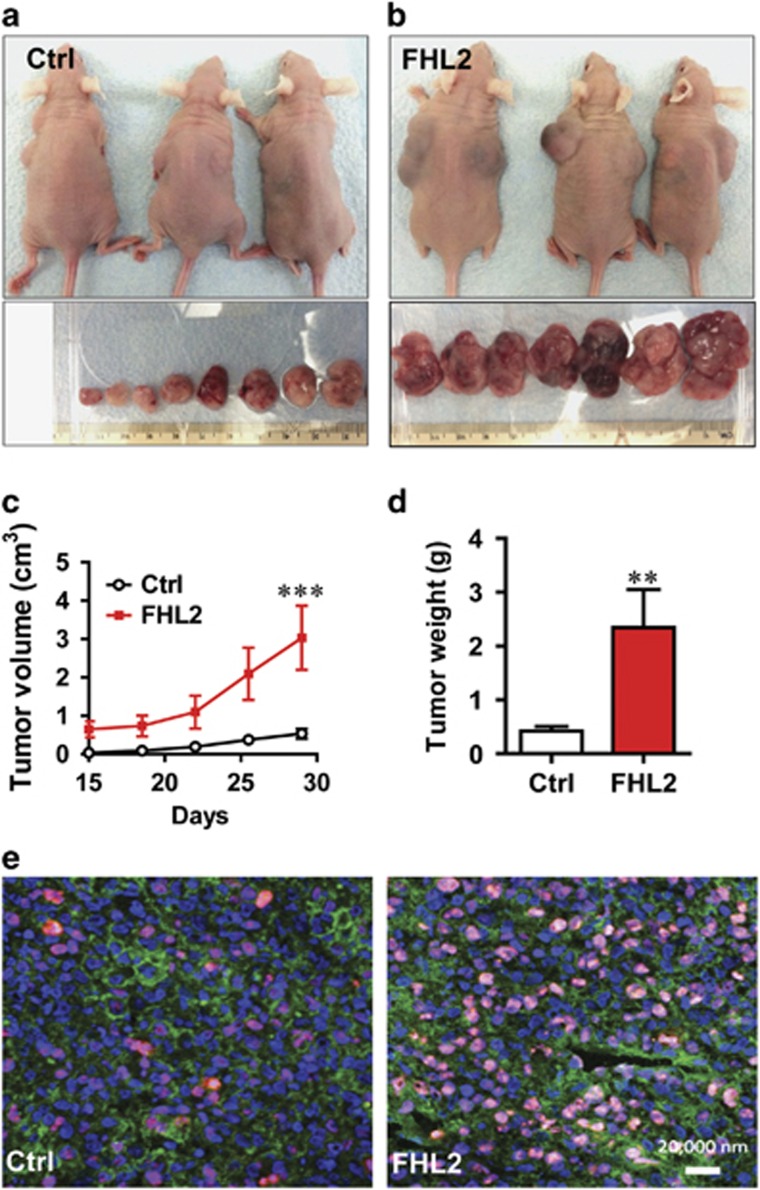
Ectopic expression of FHL2 promotes GCT tumorigenesis. (**a**) Representative images showing tumor formation in COV434 cells transfected with an empty control vector (control). (**b**) Representative images showing tumor formation in COV434 cells transfected with an FHL2-expressing vectors (FHL2). The formed tumors from control and FHL2 groups were isolated and presented under the image of each group. The minimum scale of the rule is mm. (**c**) Growth curve of tumor xenografts derived from control and FHL2-expressing COV434 cells. Each point represents mean diameter±S.E.M. of six animals. ***P<0.001 compared with control (Ctrl) on day 29. (**d**) Weights of tumor xenografts derived from control and FHL2-expressing COV434 cells. Each bar represents mean±S.E.M. of six animals. **P=0.0116 compared with control (Ctrl). (**e**) Expression of Ki67 (red) in the tumor tissues derived from control and FHL2-expressing COV434 cells. Ki67 expression was determine using fluorescent immunohistochemistry. Nuclei were stained with DAPI (blue) and actin was stained with FITC-phalloidin (green). Scale bar: 20 *μ*m

To confirm the physiological relevance of this finding, COV434 cells transfected with empty control vector (Ctrl) and COV434 cells transfected with FHL2-expressing vector were implanted subcutaneously into athymic nude mice. As expected, both cells lines formed tumors in athymic nude mice (Figures 4a and b). Tumor growth curves showed that tumors derived from the FHL2-expressing cells grew significantly faster than the control group (Figure 4c). Twenty-nine days after tumor cell implantation, the average tumor volume in the FHL2 group was approximately six times larger than that of control (Figure 4c). Similarly, the average tumor weight in the FHL2-expressing group was approximately six times more than that of control groups (Figure 4d). Consistent with the higher growth rate of tumors derived from FHL2-expressing cells, more Ki67-positive cells were present in FHL2-expressing tumors in comparison with the control tumors (Figure 4e).

### FHL2 regulates GCT cell growth and survival through regulating AKT expression

**Figure 5:**
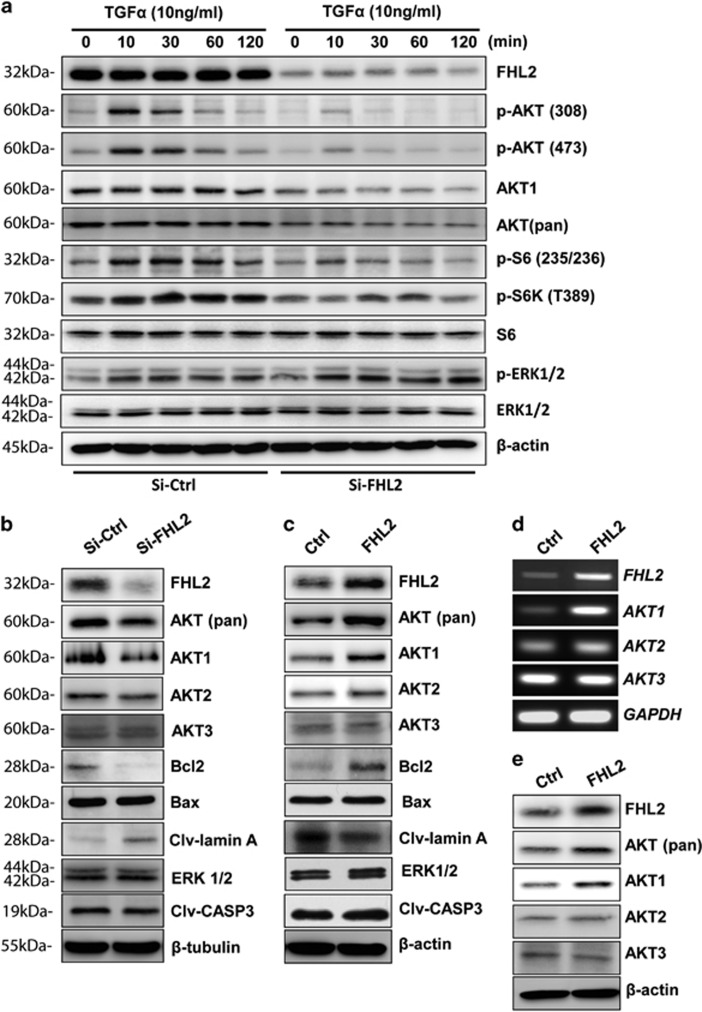
FHL2 regulates AKT1 expression in KGN cells. (**a**) Knockdown of FHL2 compromises TGF*α*-induced activation of PI3K/AKT pathway. Phosphorylated and total protein levels were determined using western blot with samples collected in three independent experiments. (**b**) Knockdown of FHL2 using FHL2 siRNA (siFHL2) reduced pan-AKT, AKT1 and BCL2 protein levels, and increased the cleaved lamin A protein level. (**c**) Ectopic expression of FHL2 increases Pan-AKT, AKT1 and other pro-survival factors. Protein expression and kinase activation were determined using western blot with samples collected from three independent samples and representative images are presented. (**d**) RT-PCR results showing that ectopic expression of *FHL2* increases *AKT1* transcription, but has no significant effect on *AKT2* and *AKT3* transcripts in the COV434-derived tumors. *GAPDH* was used as an internal control. (**e**) Representative images showing that ectopic expression of FHL2 increases pan-AKT and AKT1 protein levels *in vivo*. Protein levels were determined using western blot with samples collected from six tumors. *β*-Actin was used as a protein loading control

Our previous report indicated that TGF*α*, via regulating PI3K/AKT pathway, stimulates GCT progression.^35^ Knockdown of FHL2 in KGN cells nearly eliminated TGF*α*-induced activation of PI3K/AKT pathway, which was indicated by inhibition of the phosphorylation of AKT, S6K and S6 after treatment of FHL2-knockdown KGN cells with TGF*α* (Figure 5a). However, knockdown of FHL2 did not affect TGF*α*-induced phosphorylation of ERK1/2 in KGN cells. This suggests that FHL2 may selectively interact with the AKT pathway to regulate GCT cell growth and survival. Intriguingly, we found that knockdown of FHL2 in KGN cells not only decreased TGF*α*-induced AKT phosphorylation, but also significantly reduced AKT protein levels (Figure 5a). Further studies showed that knockdown of FHL2 selectively decreased expression of AKT1, but not AKT2 and AKT3, in KGN cells (Figure 5b). We also observed that ectopic expression of FHL2 in KGN cells increased pan-AKT and AKT1 protein levels, but had no significant effect on AKT2 and AKT3 protein levels (Figure 5c). RT-PCR results also indicated that knockdown of FHL2 in KGN cells resulted in a significant decrease in *AKT1* mRNA, but not *AKT2* and *AKT3* mRNAs (Supplementary Figure S7a), suggesting that FHL2 is a critical regulator of AKT1 transcription. FHL2 regulation of *AKT1* gene transcription was also confirmed in COV434 cells. Ectopic expression of FHL2 in COV434 cells induced significant increase in pan-AKT and AKT1 protein levels, but did not affect AKT2 and AKT3 protein levels (Supplementary Figure S8a). RT-PCR analysis also confirmed the selective increase in *AKT1* mRNA levels in COV434 cells after ectopic expression of FHL2 (Supplementary Figure S8b). Effect of FHL2 on the expression of AKT1 was also evidenced by the endogenous AKT1 levels in KGN and COV434 cells. RT-PCR results clearly showed that *AKT1* mRNA levels in KGN cells (high level of endogenous *FHL2*) is much greater than in COV434 cells that have low level of endogenous FHL2 (Supplementary Figure S7b). Finally, FHL2 regulation of AKT1 expression is supported by our *in vivo* study. *AKT1* mRNA levels in the tumor tissues derived from FHL2-overexpressing COV434 cells were significantly higher than that in tumor tissues derived from control COV434 cells (Figure 5d and Supplementary Figure S8c). Consistent with mRNA results, AKT1 protein levels in the tumor tissues derived from FHL2-overexpressing COV434 cells were significantly higher than that in tumor tissues derived from control COV434 cells (Figure 5e).

### AKT1 rescues FHL2 knockdown-induced GCT cell phenotype

**Figure 6:**
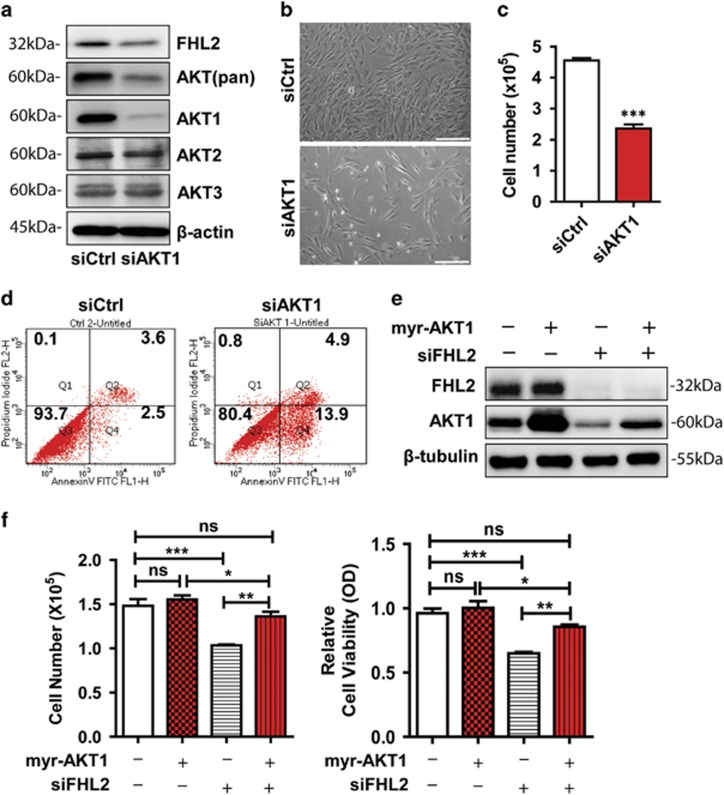
Ectopic expression and activation of AKT1 rescue FHL2 knockdown-induced arrest of KGN cell growth. (**a**) AKT protein levels in KGN cells transfected with non-targeting control siRNA (siCtrl) and AKT1 siRNA (siAKT1). Protein levels were determined by western blot. (**b**) Morphology of KGN cells transfected with non-targeting control siRNA (siCtrl) or AKT1 siRNA (siAKT1). Scale bar: 200 *μ*m. (**c**) Quantitative data showing that knockdown of AKT1 suppressed growth of KGN cells. Cell number was counted with an Invitrogen automatic cell counter. (**d**) Flow cytometry showed that knockdown of AKT1 in KGN cells induced KGN cell apoptosis. Cells were stained with the Annexin V-FITC/PI dual staining kit before analysis. (**e**) Representative images showing that ectopic expression of Myr-AKT1 in KGN cells increased AKT1 protein levels. FHL2 and AKT1 protein levels were determined by western blot with samples collected from three independent experiments. *β*-tubulin was used as a protein loading control. (**f**) Ectopic expression of myr-AKT1 rescued FHL2 knockdown-induced arrest of KGN cell growth (Left graph) and decrease of cell viability (lower graph). Cell number and viability were analyzed 5 days after cell seeding. Each bar represents mean±S.E.M. (*n*=5). ns, not significant. *P<0.05; **P<0.01; ***P<0.001

The above results indicate that FHL2 may regulate GCT cell growth via controlling the AKT1 protein level. As expected, knockdown of AKT1 in KGN cells with AKT1-specific siRNA significantly reduced cell growth (Figures 6a–c, *P*<0.001), and induced cell morphological changes similar to that observed in FHL2-knockdown KGN cells (Figures 6b and 2c). Moreover, knockdown of AKT1 significantly reduced KGN cell viability (Figure 6d and Supplementary Figure 9a, *P*<0.001), and inhibited KGN cell migration (Supplementary Figures S9b and c). Clearly, knockdown of AKT1 and FHL2 result in similar cellular phenotypes, supporting the concept that FHL2 regulating GCT cell activity via controlling AKT1 protein levels.

To further investigate the role of AKT1 in mediating the biological action of FHL2, we designed experiments to rescue AKT1 protein levels in FHL2-knockdown KGN cells. Transfection of normal KGN cells with myr-AKT1 successfully increase AKT1 protein levels (Figure 6e), but had no obvious effect on cell proliferation and viability in 3 days. However, transfection of FHL2-knockdown KGNs with myr-AKT1 completely abolished FHL2 knockdown-induced arrest of cell growth and reduction of cell viability (Figure 6f). These results convincingly suggested that FHL2 regulates GCT cell growth and viability via regulating AKT1 expression.

### FHL2 acts as a co-activator of NF*κ*B and AP-1 transcription factors to regulate AKT1 expression

**Figure 7:**
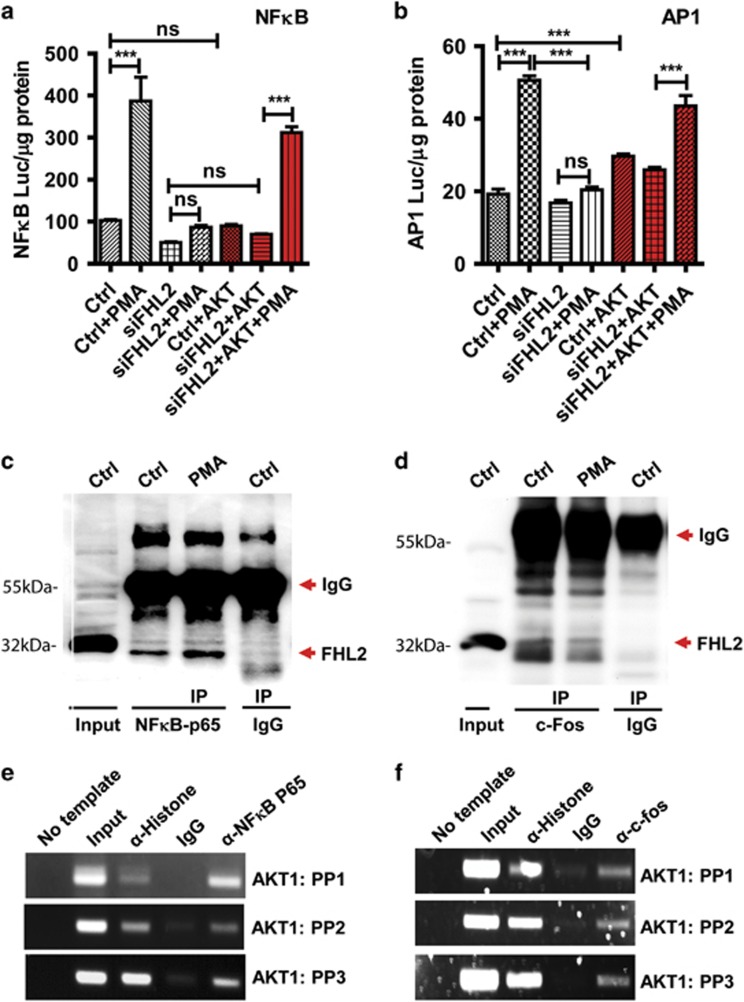
FHL2 interacts with NF*κ*B and AP-1 to drive AKT1 expression. (**a**) Knockdown of FHL2 totally blocked PMA-induced NF*κ*B-luciferase activity. Ectopic expression of myr-AKT1 rescued FHL2 deficiency-induced inhibition in NF*κ*B promoter activation in response to PMA stimulation. Relative activities of luciferase were normalized with total protein levels. (**b**) Knockdown of FHL2 eliminated PMA-induced AP-1-luciferase activity. Ectopic expression of myr-AKT1 rescued FHL2 deficiency-induced elimination of AP-1 promoter activation in response to PMA stimulation. Relative activities of luciferase were normalized with total protein levels. Each bar in (**a**) and (**b**) represents mean±S.E.M. (*n*=5). NS, not significant. *P<0.05; **P<0.01; ***P<0.001. (**c**) Co-immunoprecipitation assay showing the direct interaction of FHL2 with the NF*κ*B transcription factor. Note that the PMA treatment induced increased FHL2 binding to NF*κ*B. (**d**) Co-immunoprecipitation assay showing the interaction of FHL2 on the AP-1 transcription factor. (**e**) Chromatin immunoprecipitation (ChIP) assay showing that *AKT1* gene is the direct target of NF*κ*B transcription factor, which is associated with FHL2 as indicated in (**c**). Acetyl histone H3 was used as a positive control, while samples from the IgG group (antibody replaced with the same amount of IgG) was used as a negative control. Experiment was repeated for three times and representative images were presented. (**f**) ChIP assay showing that *AKT1* gene is the direct target of AP-1 transcription factor (c-fos), which is associated with FHL2 as indicated in (**d**). Positive and negative controls, as well as the repeats of this experiments are the same as in (**e**)

Bioinformatics analysis of the proximal promoter region of the *AKT1* gene sequence indicated that there are three NF*κ*B-binding sites and three AP-1-binding sites in the *AKT1* gene (Supplementary Figure S10). This indicates that NF*κ*B and AP-1 are potential transcription factors that interact with FHL2 to regulate *AKT1* gene expression. Therefore, we first used luciferase-based promoter reporter assays to examine the role of FHL2 in NF*κ*B and AP-1 gene transcription activity. We found that in the KGN cells, PMA treatment induced significant increase in NF*κ*B and AP-1 transcription activities, as indicated by the significant increases in NF*κ*B and AP-1 promoter-driven luciferase in luciferase assays (Figures 7a and b). Knockdown of FHL2 totally abolished PMA-induced increase in NF*κ*B and AP-1 transcription activity. However, transfection of FHL2-knockdown KGN cells with myr-AKT1 vector recovered PMA action on the NF*κ*B and AP-1 transcription activities (Figures 7a and b). Clearly, NF*κ*B and AP-1 are two important transcription factors that are involved in FHL2 regulation of AKT1 gene expression.

We then performed co-immunoprecipitation assays (Co-IP) to determine whether FHL2 interacts with NF*κ*B and/or AP-1 transcription factor(s) in GCT cells. The Co-IP analysis showed the presence of FHL2 in NF*κ*B P65 immunoprecipitates, suggesting that FHL2 may function as a co-activator of NF*κ*B transcription factor (Figure 7c). Similar, FHL2 was identified in c-Fos immunoprecipitates, suggesting that FHL2 also acts as a co-activator of AP-1 transcription factor (Figure 7d). Finally, we used chromatin immunoprecipitation (CHIP) assay to examine whether NF*κ*B and AP-1 interact with *AKT1* gene. The successful amplification of *AKT1* from NF*κ*B P65 and c-Fos pull-downs using six different primer pairs convincingly indicated that NF*κ*B and AP-1 directly bind to *AKT1* gene (Figures 7e and f).

## Discussion

Previous studies indicate that the role of FHL2 in cell proliferation is tissue and cell type specific.^28, 36, 37, 38^ For example, FHL2 inhibits anchorage-dependent and -independent growth of hepatocellular carcinoma cells through a TGFß-like signaling pathway.^28^ On the other hand, FHL2 acts as a TGF*β*1-responsive gene and promotes colon cancer cell adhesion, migration and invasion.^36^ Although FHL2 expression in the normal ovary^32^ and ovarian cancer^31^ has been reported, its role in the ovarian tumorigenesis has not been reported. In the present study, we found that FHL2 is overexpressed in the GCT tissues. By manipulating FHL2 expression in KGN cells and COV434 cell, our data indicate that FHL2 acts as a GCT tumor cell growth-promoting factor, implying that FHL2 may function as an oncogenic protein in ovarian tumors. Overexpression of FHL2 in epithelial ovarian cancer has been reported.^31^ Our recent studies showed that overexpression of FHL2 in SKOV3 cells greatly promotes cell proliferation, while knockdown of FHL2 in SKOV3 cells using FHL2 shRNA induced drastic cell death (Hua *et al.*, unpublished observation), suggesting that FHL2 also acts as a oncogenic protein in epithelial ovarian cancers. Further studies are warranted to provide more direct and systematic evidence on the role of FHL2 in the initiation and progression of epithelial ovarian cancer.

GCT is able to metastasize into a variety of organs and tissues.^5, 39^ Extraovarian disease spread is associated with higher GCT-related mortality.^39^ The molecular mechanisms underlying GCT metastasis are unclear. In the present study, we provide evidence that FHL2 affects not only GCT cell growth but also cell migration. Consistent with our observations, previous studies also indicated that FHL2 facilitates cell migration in osteosarcoma and glioblastoma.^27, 40^ Moreover, our observations are consistent with results from FHL2-deficient mice, in which cutaneous wound healing was impaired.^41^ Since the contribution of tumor cell motility to metastasis has been well documented,^42^ overexpressed FHL2 in GCTs may also contribute to the metastasis of human GCT. Although the exact mechanism by which FHL2 regulates GCT migration is unclear, we observed that FHL2 plays a functional role in the regulation of dynamic structure of cellular cytoskeleton. We found that knockdown of FHL2 resulted in the re-arrangement of actin filaments, leading to the assembly of cell stress fibers. Since long and thick stress fibers are associated with reduced cell motility,^43^ FHL2 may regulate GCT cell migration through regulating cellular cytoskeleton.

The mechanism by which FHL2 regulates the ovarian tumor cell growth is also unclear. It is known that GCT cells are derived from granulosa cells.^2^ FSH is the most important hormone for granulosa cell survival, growth and proper function.^44^ Importantly, recent studies have shown that the PI3K/AKT pathway is a pivotal signaling corridor necessary for transducing the FSH signal.^45^ Our previous studies also indicated that TGF*α* and the Hippo pathway are key regulators of GCT cell growth and survival.^11, 35^ Intriguingly, the PI3K/AKT pathway is a key mediator of TGF*α* and YAP actions in ovarian tumor cells.^11, 35, 46^ Consistent with these findings, combined comparative genomic hybridization and transcriptomic analyses of GCTs identified AKT1 as one of the most potential genes whose alterations might contribute to GCT initiation and progression.^47^ Collectively, these studies point towards the AKT pathway as a major regulator of GCT cell growth and survival. In the present study, we provide evidence that FHL2 directly affects the AKT signaling pathway. We found that ectopic expression of FHL2 in GCT cells induced significant increases in *AKT1* mRNA and protein levels in both *in vitro* and *in vivo* models. Knockdown of FHL2 eliminated TGF*α*-induced activation of AKT and suppressed AKT1 expression. Knockdown of AKT1 in GCT cells suppressed cell growth, increased cell apoptosis and induced the appearance of elongated fibroblast-like cells, which mimicked the phenotype observed in FHL2-knockdown KGN cells. Most importantly, ectopic expression of AKT1 in GCT cells rescued FHL2 knockdown-induced suppression of cell growth and reduction of FHL2 downstream transcription activity. These studies clearly indicate that FHL2 may regulate GCT cell growth and survival via controlling *AKT1* transcription, and subsequently, the PI3K/AKT signaling pathway.

Evidence from genetically modified animal models indicates that the three AKT isoforms (AKT1, AKT2 and AKT3) have different functions. Akt3 plays a important role in brain development,^48^ while Akt2 is critical in the maintenance of glucose homeostasis.^49, 50^ Akt1 knockout mice are smaller in size and Akt1-null cells display higher rates of apoptosis, indicating a critical role for Akt1 in cell growth and survival.^49, 51^ Simultaneous deletion of Akt1 and Akt2 causes lethality shortly after birth,^52^ while simultaneous deletion of Akt1 and Akt3 results in embryonic lethality.^53^ However, mice with a single functional allele of Akt1 (Akt1^+/−^Akt2^−/−^Akt3^−/−^) are viable despite reduced body weight and insulin and glucose intolerance.^54^ These results indicated Akt1 is essential for normal cell growth and is sufficient to perform all critical Akt functions in postnatal survival. Our data indicated that knockdown of FHL2 suppressed expression of AKT1 (but not AKT2 and AKT3), inhibited cell growth and increased GCT cell apoptosis, while ectopic expression of FHL2 induced AKT1 expression, increased GCT cell survival and stimulated GCT cell growth. Owing to the importance of AKT1 in cell growth and survival, and the fact that FHL2 regulates AKT1 expression, we believe that FHL2 is critical for the viability and growth of GCT cells. FHL2 represents a very promising target for the development of novel drug for the GCT treatment.

Over the last 10 years, numerous studies have elucidated the basic mechanisms underlying the activation of the AKT kinase. However, few studies have focused on the transcriptional regulation of AKTs. In the present study, we indicated that FHL2 is a key regulator of AKT1 gene expression, at least in the ovarian tumor cell lines. *In silico* analysis of *AKT1* gene showed that several transcription factors have potential to bind to the promoter of *AKT1* gene, including NF*κ*B, AP-1, Stat3 and GLI1. Since NF*κ*B and AP-1 interact with both FHL2 and AKT1, we hypothesize that FHL2 may employ NF*κ*B and AP-1 transcription factors to regulate *AKT1* gene transcription. Results from our Co-IP and ChIP analyses clearly indicated that FHL2 interacts with NF*κ*B and AP-1 transcription factors to drive the transcription of *AKT1* gene in GCT cells.

**Figure 8:**
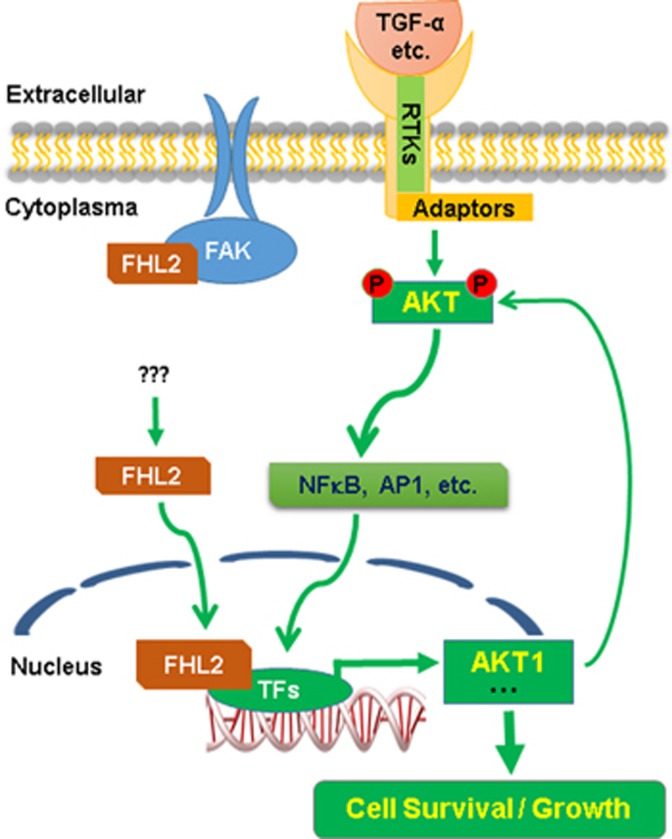
A schematic diagram showing the proposed mechanism for FHL2 to regulate GCT cell growth. FHL2 in the GCT tissues is overexpressed with an up to date unknown mechanism(s). Overexpression of FHL2 in GCT tissues interacts with NF*κ*B and AP-1 transcription factors to promote the expression AKT1. Increased AKT1 in GCT cells drives the downstream genes to increase GCT cell viability and promote tumor cell growth. Moreover, our previous data have shown that GCT tumor cells produce TGF-*α* and EGF, and express all four EGFR isoforms. TGF*α* and EGF, via EGFRs, may activated PI3K/AKT pathways, leading to accelerated GCT tumor cell survival and promote GCT tumor progression

In conclusion, our study demonstrates that FHL2 is overexpressed in human GCT tumors. Overexpression of FHL2 stimulates GCT cell growth, enhances GCT cell survival and promotes GCT tumorigenesis. Importantly, we found that FHL2 promotes GCT tumorigenesis via activating NF*κ*B- and AP-1-driven *AKT1* gene transcription. Increased AKT1 (induced by overexpression of FHL2) would in turn promotes the activation of NF*κ*B and AP-1, forming a positive-feedback loop to drive GCT tumor malignant progression (Figure 8). Our previous studies have indicated that GCT tumor cell expresses all four EGFR isoforms (EGFR, ERBB2, ERBB3 and ERBB4) and is able to produce EGFR ligands such as EGF and TGF-*α.*^35^ We also found that TGF-*α* is able to promote GCT tumor cell growth via a TGF*α*/ERBBs autocrine/paracrine loop in GCT.^35^ AKT1 is a known critical mediator of the EGFR signaling transduction. FHL2-induced increase in AKT1 may also be involved in mediating TGF-*α*/ERBBs autocrine/paracrine loops in GCT tumor cells. Therefore, FHL2 is a key regulator of GCT tumor cell survivor and GCT progression, and is also a very promising target for development of drugs against ovarian granulosa cell tumors.

## Material and Methods

### Cell lines and human GCT tissue slides

The adult GCT cell line KGN was from Riken Biosource Center (Ricken Cell Bank, Ibaraki, Japan). The KGN cell line is an ideal cellular model for studying the growth and metastasis of the adult GCTs.^2^ The COV434 cell line was from Dr C E van der Minne (University Hospital, Leiden, Netherlands) and is an ideal cellular model for studying FHL2 function because of its very low endogenous FHL2 expression. Cell lines used in this study were passaged less than 12 times in our laboratories and were validated for their authenticity with short tandem repeat (STR) analysis performed by both the Riken Biosource Center (Ricken Cell Bank) and the Genetica DNA Laboratories (Burlinton, NC, USA). Formalin-fixed, paraffin-embedded normal human ovarian tissues (*n*=10) and human GCT (*n*=12) slides were from the Department of Pathology, Tianjin Medical University Cancer Hospital. The retrospective use of these human tissue slides was permitted by protocols approved by the UNMC Institutional Review Board and Tianjin Medical University Institutional Review Board.

### Immunohistochemistry analysis of FHL2 expression in ovarian tissues

The expression of FHL2 protein in paraffin-embedded human ovarian tissues and GCTs was detected by a previously described peroxidase-based immunohistochemistry protocol.^35^ Immunosignals were visualized with a DAB kit (Invitrogen, Grand Island, NY, USA). The sections were counterstained with Mayer's hematoxylin and scanned with an iSCAN Coreo Slide Scaner (Ventana Medical Systems, Inc., Oro Valley, AZ, USA). The positivity (i.e., the number of positively stained cells relative to the total number of cells in the tissue section) and the intensity of the positive immunosignals were quantified with Aperio ImageScope software (Leica Biosystems Inc., Buffalo Grove, IL, USA).

### Fluorescent immunocytochemistry

Frozen sections at 6 *μ*m were stained with a protocol established in our laboratory.^11^ Images were captured using a Zeiss 710 Meta Confocal Laser Scanning Microscope and analyzed using the Zeiss Zen 2010 software (Carl Zeiss Microscopy, LLC, Thornwood, NY, USA).

### RT-PCR and western blot analysis

Control and treated cells were harvested on ice with ice-cold cell lysis buffer. Protein levels were determined using western blot with a protocol established in our laboratory.^11^ The immunosignal was detected using a SuperSignal West Femto Chemiluminescent Substrate Kit (Pierce/Thermo Scientific, Rockford, IL, USA). The images were captured and analyzed with a UVP gel documentation system (UVP; LLC, Upland, CA, USA). Total RNA was extracted by RNeasy Mini Kit (Qiagen, Valencia, CA, USA), and cDNA was prepared using SuperScript First-Strand kit (Life TechnlogyTM, Grand Island, NY, USA). RT-PCR was performed with a protocol established in our laboratory.^55, 56^ The primers used in our study have been validated previously.^57, 58^

### Establishment of stable cell lines with ectopic expression of FHL2

KGN and COV434 cells were cultured to 30–40% confluence and then transfected with lentivirus-based human FHL2 expression and control constructs. Vectors (LV160802, LV590) were commercially purchased from Applied Biological Materials, Inc. (Richmond, BC, Canada). Two days following transfection, cells were selected with puromycin (0.3–0.5 ng/ml) for 7 days. Stable cell lines were examined for FHL2 expression by immunoblotting and RT-PCR.

### Cell proliferation assay

To determine the effect of FHL2 on GCT cell proliferation, KGN cells were plated in six-well culture plates and incubated in a growth medium supplemented with 10% FBS until 60% confluent. The cells were then transfected with non-targeting siRNA as a negative control or FHL2 siRNA for 6 h using lipofectmine RNAimax (Invitrogen) according to the manufacturer's instruction. The cells were harvested 72 h after siRNA transfection for determination of protein levels or cell numbers. FHL2 protein expression was determined by western blot analysis and the cell numbers were quantified with an Invitrogen Countess automated cell counter. The effect of FHL2 on GCT cell proliferation was also determined in KGN cell lines that overexpress FHL2 proteins. Cell number was counted every other day.

### Cell viability analysis

MTT assays were performed to determine whether alterations in FHL2 and AKT1 expression were associated with changes in cell viability. KGN cells transfected with control vectors, FHL2-expressing vectors, non-targeting control siRNA, FHL2 siRNA or AKT1 siRNA were incubated in growth medium for 72 h before performing the 3-(4,5-dimethylthiazol-2-yl)-2,5-diphenyltetrazolium bromide (MTT) assay using a protocol established in our laboratory.^55, 56^

### Cell cycle and apoptosis analysis

Cell cycle and apoptosis analysis was performed by flow cytometry. Control and treated cells were trypsinized, fixed and permeabilized as described previously.^11^ The cells were then labeled with propidium iodide for 30 min at 37 °C and flow cytometry was used to determine the cell-cycle distribution of the KGN cells. Apoptosis was analyzed by cell surface presence of Annexin V using the Annexin V-FITC/PI or Annexin V-APC/PI Dual Staining Apoptosis Assay Kit as described by the manufacturer (BioVision, Inc., Milpitas, CA, USA).

### Cell migration assay

A chemotaxis assay was used to determine the effect of FHL2 on KGN cell migration. KGN cells (4 × 10^5^) in 250 ml of serum-free DMEM were placed in a Transwell insert (8 mm pore size; Corning-Costar, Lowell, MA, USA). The inserts were then placed in the wells of a 24-well-plate containing 750 ml of DMEM-FBS (5%) and incubated at 37 °C for 6 h. After incubation, the cells on the top of the membrane were removed with a cotton swab. The cells migrated to the underside of the membrane were fixed and stained with 0.04% crystal violet in methanol for 30 min. Cells were then photographed (10 × 10magnification) and 10 pictures per group were quantified under a microscope.

The wound-healing assay was also used to determine whether FHL2 regulates KGN cell motility. KGN cells were cultured in six-well cell plates until confluent. Wounds were made by scratching the cell layer with a 100-*μ*l pipette tip. After washing away the cell debris, pictures were taken for each ‘wound' with an Olympus inverted microscope equipped with a DP71 digital camera (Olympus America, Inc., Center Valley, PA, USA). The cells were incubated in serum-free medium for 20 h and then another picture was taken for each ‘wound'.

### Cell transformation assays

Cell transformation and anchorage-independent growth assays were performed using a fluorescence-based CytoSelect 96-well cell transformation assay kit (Cell Biolabs, Inc., San Diego, CA, USA). KGN cells were plated in soft agar in a 96-well plate at 5000 cells/well and cultured for 20 days. COV434 cells were plated at 10 000 cells/well and incubated for 7 days. Cell transformation was determined according to the protocol provided by the manufacturer. Colony number was counted under a light microscope. The relative fluorescence unit (RFU) was determined with a BMG Fluostar Optima Microplate reader (BMG LABTECH GmbH, Ortenberg, Germany).

### Luciferase assays

Luciferase assays were conducted by plating 5 × 10^4^ cells per 12-well plate. Cells were transfected with non-targeting control siRNA (negative control) or siFHL2 siRNA for 24 h before infected with adenovirus vectors of AP-1 or NF*κ*B. Phorbol myristae acetate (PMA, 20 nM) was used as a positive control. Cells were lysed in 1 × passive lysis buffer and luciferase activity was measured using the Luciferase Reporter Kit (Promega, San luis Obispo, CA, USA). All luciferase data were normalized with total protein levels and was presented as ratio relative to the relevant control cells.

### Tumorigenicity in nude mice

Control COV434 cells and COV434 cells with stable ectopic expression of FHL2 were trypsinized and collected. Cell suspensions (10^7^cells/100*μ*l of DMEM) were mixed with 100 *μ*l of Matrigel BD Bioscience (San Jose, CA, USA) and inoculated subcutaneously into the shoulder (left side and right side) of 5-week old female athymic nude mice (Harlan Sprague Dawley). The use of animal was approved by the Institutional Animal Care and Use Committee (IACUC) at the University of Nebraska Medical Center. Mice were maintained under a 12 h light/ 12 h dark cycle with free access to water and standard mouse diet. Tumor size was measured 2 weeks after initial inoculation. The volumes of tumors were calculated as follows: *V*=*R*1^2^ × *R*2^2^ × 3.142/6, where *R*1 and *R*2 are the short and long diameters of the tumors, respectively.

### AKT1 rescue analysis

The adenoviruses expressing constitutively active Akt1 (Ad-myr-Akt1) and *β*-galactosidase (Ad-*β*-gal) were a generous gift from Dr. Kenneth Walsh (Tufts University School of Medicine, Boston, MA, USA). The structure and effectiveness of adenoviral constructs have been described before.^59, 60^ KGN cells were grown in complete DMEM medium to 60–70% confluence in 12-well plates and were infected with Ad-myr-AKT1 or Ad-*β*-gal 24 h after seeding. FHL2 siRNA or siRNA negative control (siGLO) was then transfected 36 h after seeding. Cells were then cultured at DMEM growth medium for another 3 days. Total AKT and FHL2 protein levels were detected by western blot. Cell number, cell viability and luciferase activity were detected as described above.

### Co-IP and ChIP assay

Control and treated and KGN cells were washed five times with ice-cold PBS and lysed in RIPA buffer on ice. The cell lysates were collected, sonicated and pre-cleaned with an irrelevant rabbit antibody. Pre-cleared cell lysates were then incubated overnight with rabbit antibodies against NF*k*B p65, c-Fos (Cell Signaling Technology Inc., Danvers, MA, USA), or normal rabbit IgG (Santa Cruz, Dallas, TX, USA). Twenty microliters of protein A magnetic beads (Santa Cruz) were added to the mixture and incubated for 30 min at 4 °C. The beads were then pelleted by centrifugation and washed for five times with cell lysis buffer. The pull-down proteins were fragmented by western blot using a protocol described before^11^ and probed with an FHL2 antibody. The total KGN cell lysates were used as input.

Chromatin immunoprecipitation was performed using an EZ Chromatin Immunoprecipitation Assay Kit with a protocol described by the manufacturer (EMD Millipore, Billerica, MA, USA). Pre-cleared chromatin of KGN cells were immunoprecipitated with rabbit polyclonal antibodies against NF-*κ*B p65, c-FOS (Cell Signaling Technology Inc.), normal rabbit IgG (Santa Cruz) or acetyl Histone H3 (antibody against acetyl histone H3 antibody is included in the kit). The total cell chromatin extract was used as input. Precipitated chromatin was amplified with platinum Tap DNA polymerase (Invitrogen). Three primer pairs flanking different sequences of the *AKT1* gene were used for PCR. The multiple PCR primer pairs were expected to confirm the specificity of *AKT1* amplification. The primer pairs used for c-Fos ChIP assay were: AKT1-F1: 5′-CAAAGTCCCCCTTTTGTGAG-3′ AKT1-R1: 5′-GGGGTGGCTTAGGTTGACTT-3′ AKT1-F2: 5′-ATTCACTCCTGGGTCTCTCG-3′ AKT1-R2: 5′-GCCTGCCTTTACCATAAGCA-3′ AKT1-F3: 5′-GCTGGCCTGGTGTATACGTT-3′ AKT1-R3: 5′-CAGAGGGCTGGACTCAAAGA-3′. The primer pairs used for NF-kB p65 ChIP assay were: AKT1-F4: 5′-CTGCTGCTGGGACCTACAC-3′ AKT1-R4: 5′-ACCCCAGACAGAATGGTCAG-3′ AKT1-F5: 5′-TCCTCCCTGAATTCCTTCCT-3′ AKT1-R5: 5′-GGCGTCTGGTGTCTGTCTCT-3′ AKT1-F6: 5′-AGTCTGGCCTGCTTTCCATC-3′ AKT1-R6: 5′-AAGGGTTGCTTTGCACTGATT-3′.

### Statistical analysis

All experiments were repeated at least three times unless otherwise noted. Eight mice were used in each group in the *in vivo* animal studies. Statistical analysis was conducted using GraphPad Prism software (GraphPad Software, Inc., La Jolla, CA, USA). Data were analyzed for significance of difference by one-way analysis of variance with Tukey's post-test (multiple groups) or Welch's *t*-test (two groups). A *P-*value of<0.05 was considered to be significant.

## Figures and Tables

**Table 1:** Effect of FHL2 knockdown on cell cycle progression

**Cell-cycle phases**	**Control (%)**	**siFHL2 (%)**	***P*-value**
G1	86.10±0.19	90.90±0.24	0.0006
S	10.19±0.45	5.45±0.25	0.0027
G2/M	3.71±0.28	3.64±0.15	0.8454

## Abbreviations

AKT1: V-Akt murine thymoma viral oncogene homolog 1
AP-1: activator protein 1
AR: androgen receptor
BCL2: B-cell CLL/lymphoma 2
CDT1: chromatin licensing and DNA replication factor 1
CHIP: chromatin immunoprecipitation
CREB: CAMP responsive element-binding protein
DAB: 3,3*-N*-diaminobenzidine tertrahydrochloride
DAPI: 4',6-diamidino-2-phenylindole
E4F1: E4F transcription factor 1
EGFR: epidermal growth factor receptor
ERK1/2: extracellular signal-regulated kinase 1/2
FHL2: four and a half LIM domains 2
FOXL2: Forkhead Box L2
FSH: follicle-stimulating hormone
GAPDH: glyceraldehyde-3-phosphate dehydrogenase
GCT: granulosa cell tumor
MCM2: minichromosome maintenance complex component 2
MTT: 3-(4,5-dimethylthiazol-2-yl)-2,5-diphenyltetrazolium bromide
NF*κ*B: nuclear factor of kappa light polypeptide gene enhancer in B cells
PI3K: phosphoinositide 3-kinase
PLZF: promyelocytic leukemia zinc finger
PMA: phorbol myristate acetate
RUNX1: Runt-related transcription factor 1
TGF*α*: tranforming growth factor *α*
TUBB: beta-tubulin
WT-1: Wilms tumor 1
YAP1: Yes-associated protein 1
