# Supplementary material for: The four and a half LIM domains 2 (FHL2) regulates ovarian granulosa cell tumor progression via controlling AKT1 transcription
Source: Cell Death Dis. 2016 Jul 14;7(7):e2297–. doi: 10.1038/cddis.2016.207 (PMC4973349; doi:10.1038/cddis.2016.207)
Supplement: Supplementary Figure 1 [file cddis2016207x1.pdf]

Supplementary Information

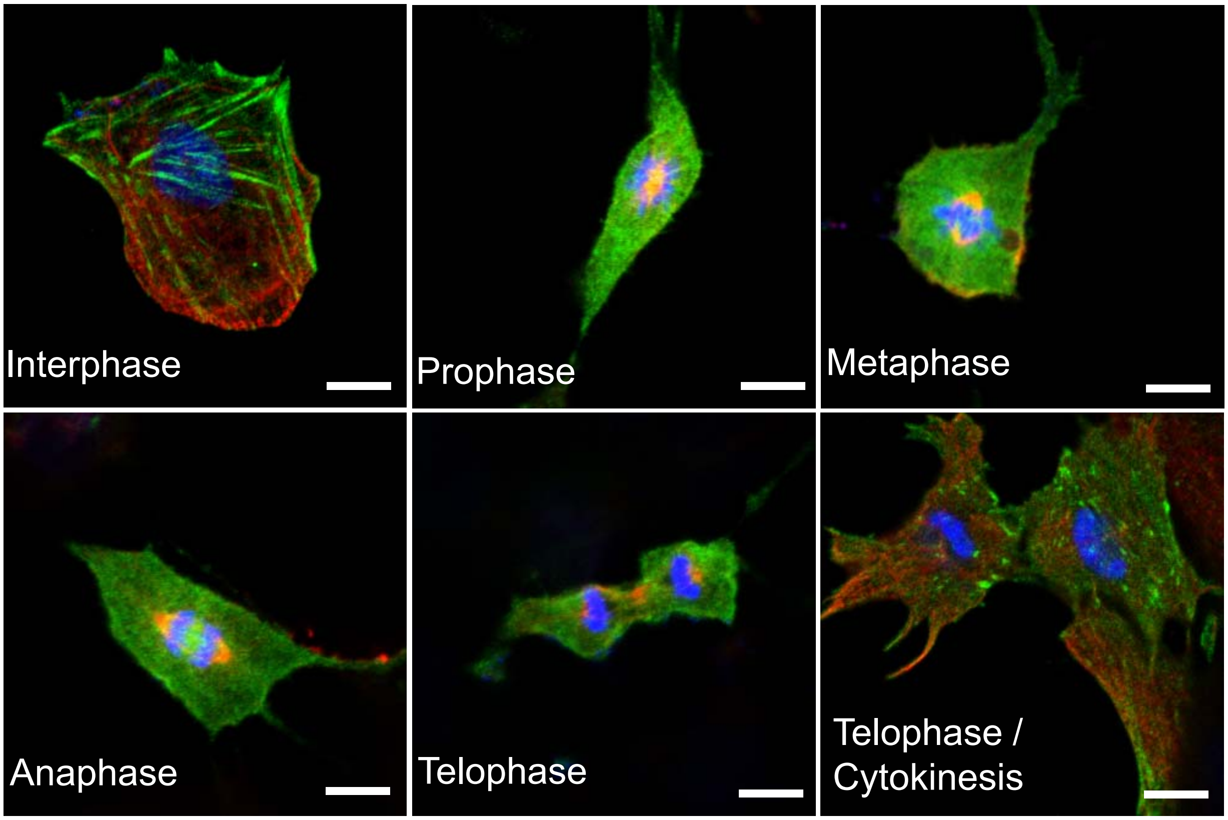

Supplementary figure S1. Cell cycle stage-dependent distribution of FHL2 protein in KGN cells. FHL2 protein expression and localization were determined by fluorescent immunohistochemistry. Images were captured with a Laser Scanning Confocal Microscope. FHL2 immunosignal was presented as green and β-tubulin was presented as red color. Nuclei were stained with DAPI (blue). Scale bar: 10μm.
