# Supplementary material for: The four and a half LIM domains 2 (FHL2) regulates ovarian granulosa cell tumor progression via controlling AKT1 transcription
Source: Cell Death Dis. 2016 Jul 14;7(7):e2297–. doi: 10.1038/cddis.2016.207 (PMC4973349; doi:10.1038/cddis.2016.207)
Supplement: Supplementary Figure 2 [file cddis2016207x2.pdf]

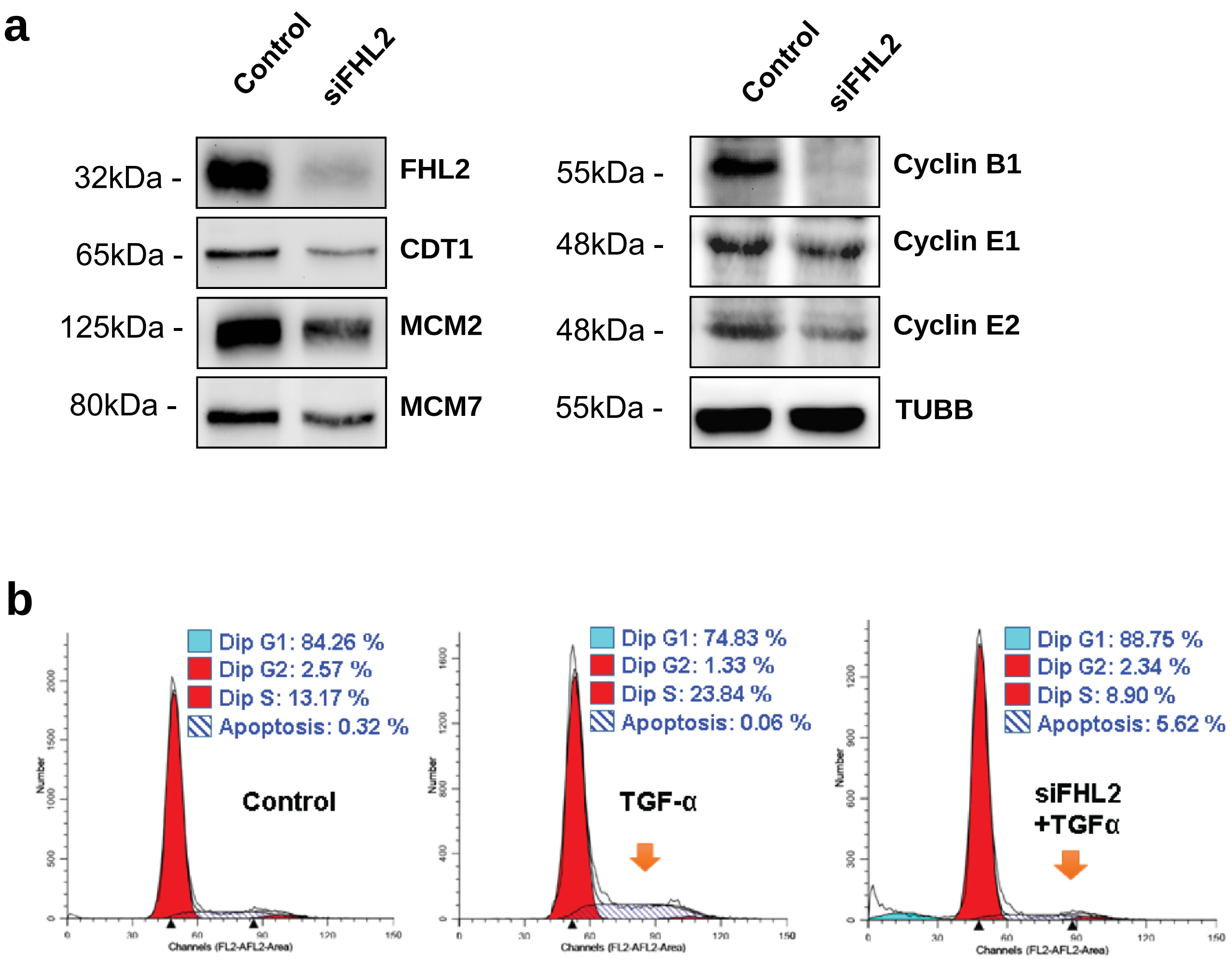

Supplementary figure S2. Effect of FHL2 on GCT cell growth. **a**) Knockdown of FHL2 suppressed expression of pro-proliferation factors. Protein levels were determined by Western blot. β-tubulin (TUBB) was used as a protein loading control. **b**) Knockdown of FHL2 in KGN cells eliminated TGFα-induced increase in cells in the S phase.
