# Supplementary material for: The four and a half LIM domains 2 (FHL2) regulates ovarian granulosa cell tumor progression via controlling AKT1 transcription
Source: Cell Death Dis. 2016 Jul 14;7(7):e2297–. doi: 10.1038/cddis.2016.207 (PMC4973349; doi:10.1038/cddis.2016.207)
Supplement: Supplementary Figure 3 [file cddis2016207x3.pdf]

Supplementary Information

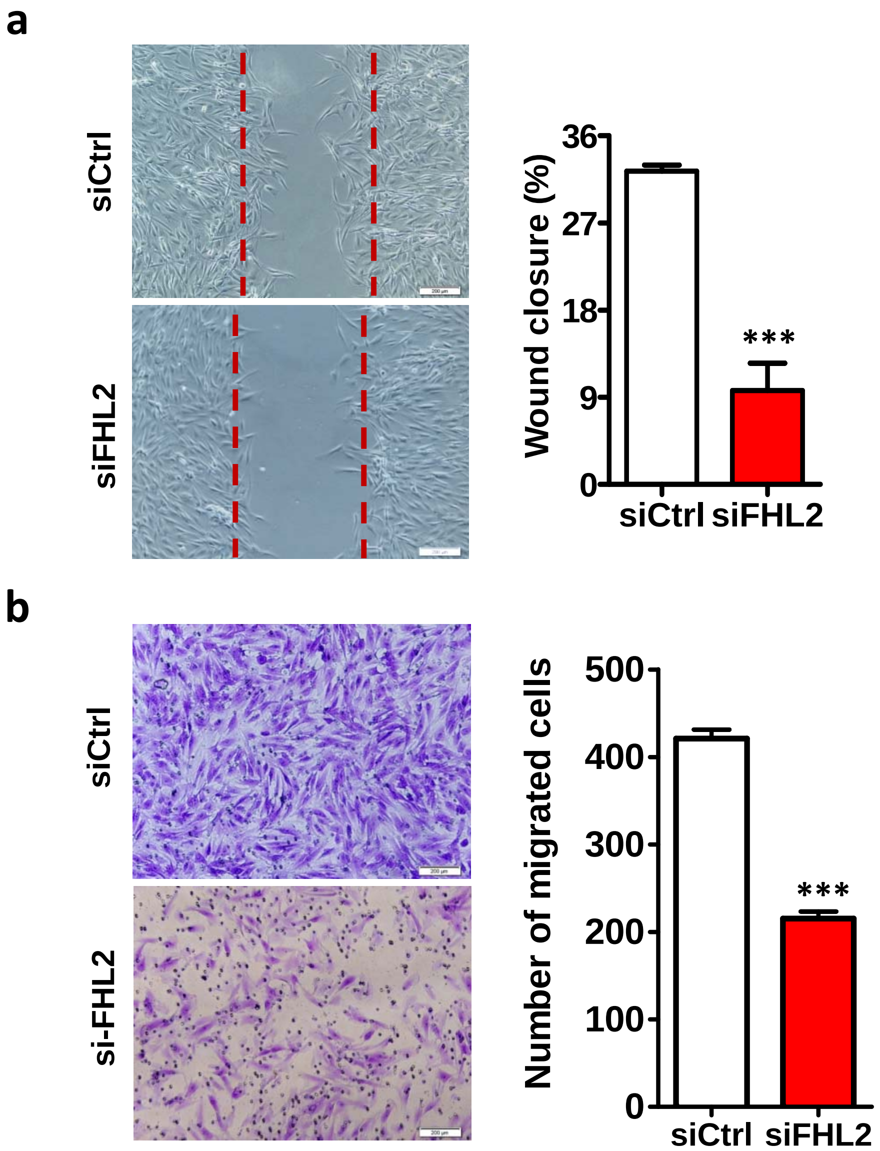

Supplementary figure S3. Knockdown of FHL2 significantly compromised KGN cell migration. **a**) Wound healing assay was performed to determine the effect of FHL2 on KGN cell migration. Left: Representative images showing the migration (or wound closure) of KGN cells transfected with non-targeting control siRNA (siCtrl) or FHL2 siRNA (siFHL2). Cells were allowed to migrate for 18h before wound closure area measurement. The initial wound edge is highlighted by red dashed lines. Scale bar: 200µm. Right: Quantitative data showing cell mobility change in KGN cells. The wound area was quantified with Microsuit^{TM} FIVE software. Each bar represents mean ± SEM (n=8). ***: *P* < 0.001 compared with control (siCtrl). **b**) Cell migration was also analyzed with a Transwell migration assay system. Left: representative images showing the migrated KGN cells transfected with non-targeting control siRNA (siCtrl) or FHL2 siRNA (siFHL2). Scale bar: 200µm. Right: Quantitative results showing changes in the cell motility of KGN cells after knockdown of FHL2. Migrated cells were counted manually under a microscope. Each bar represents mean ± SEM (n=8). ******* : *P* < 0.001, compared with siRNA control (siCtrl).

Hua *et al*, Fig. S3
