# Supplementary material for: The four and a half LIM domains 2 (FHL2) regulates ovarian granulosa cell tumor progression via controlling AKT1 transcription
Source: Cell Death Dis. 2016 Jul 14;7(7):e2297–. doi: 10.1038/cddis.2016.207 (PMC4973349; doi:10.1038/cddis.2016.207)
Supplement: Supplementary Figure 4 [file cddis2016207x4.pdf]

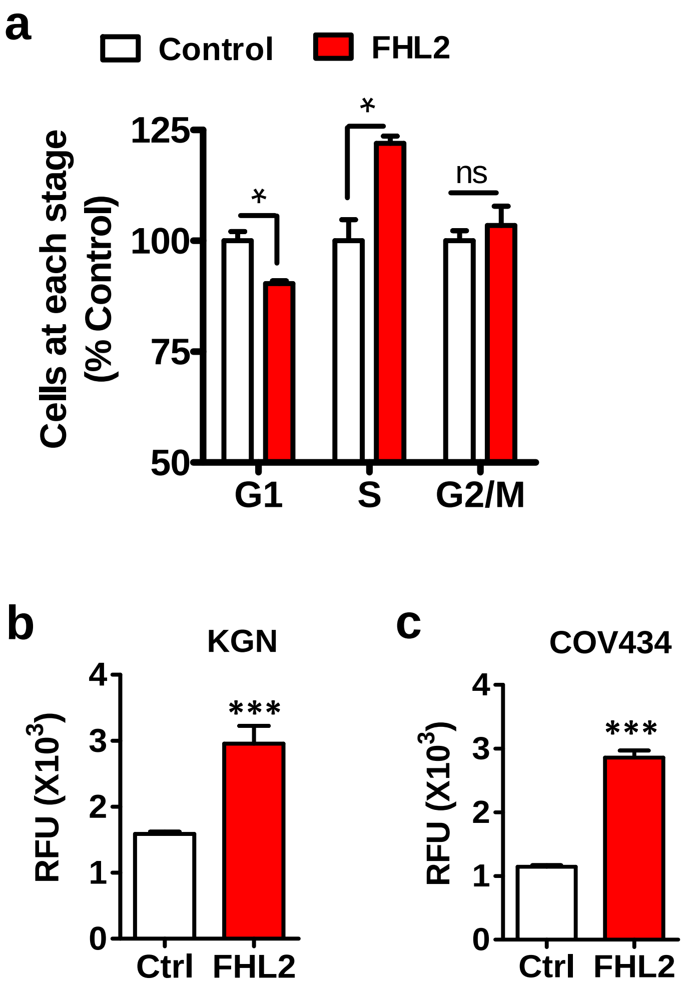

Supplementary figure S4. **Overexpression of FHL2 in KGN cells promotes cell growth and migration. a**) Overexpression of FHL2 in KGN cells promotes cell cycle progression. Each bar represents mean ± SEM of three independent assays. *: $P < 0.05$ compared with control; ns: no significant difference compared with control. **b**) Fluorescence-based quantitative soft agar assay showing the relative colony numbers (indicated by RFU) in KGN cells transfected with empty vector (Ctrl) or FHL2-expressing vector (FHL2). **c**) Fluorescence-based quantitative soft agar assay showing the relative colony numbers (indicated by RFU) in COV434 cells transfected with empty vector (Ctrl) or FHL2-expressing vector (FHL2). Each bar represents mean ± SEM of five independent repeats. ***: $p < 0.001$ compared to control group (Ctrl).
