# Supplementary material for: The four and a half LIM domains 2 (FHL2) regulates ovarian granulosa cell tumor progression via controlling AKT1 transcription
Source: Cell Death Dis. 2016 Jul 14;7(7):e2297–. doi: 10.1038/cddis.2016.207 (PMC4973349; doi:10.1038/cddis.2016.207)
Supplement: Supplementary Figure 5 [file cddis2016207x5.pdf]

Supplementary Information

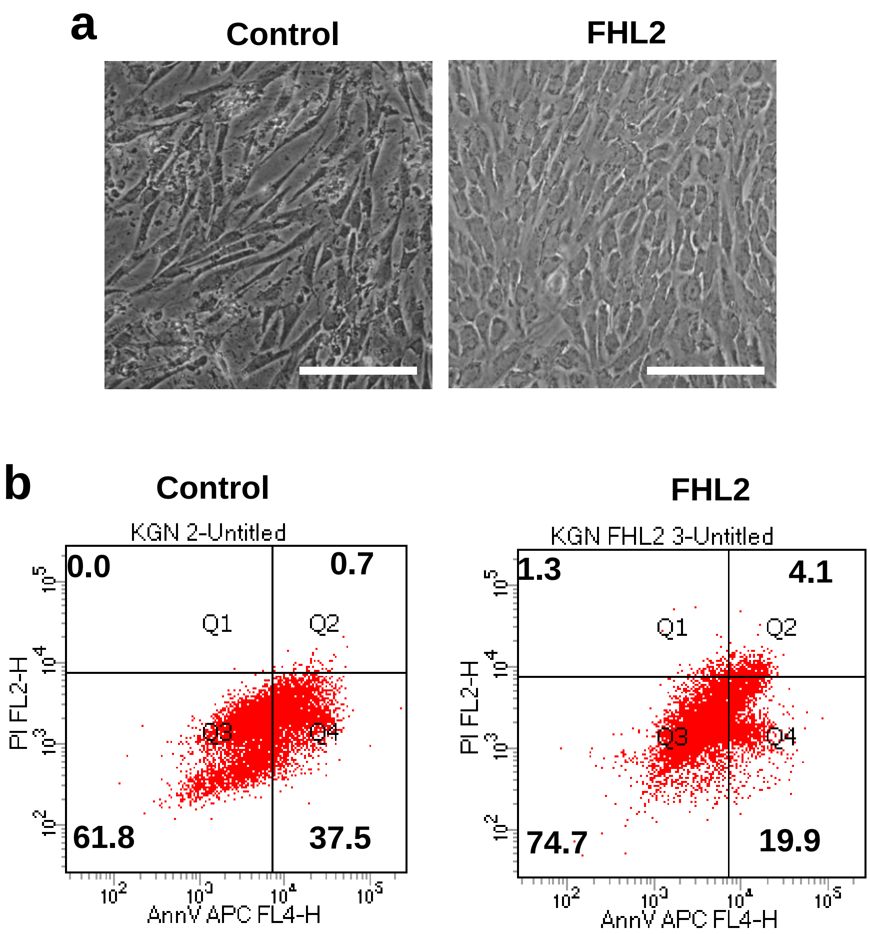

Supplementary figure S5. Ectopic expression of FHL2 in GCT cells promote their growth and viability. a) Representative images showing morphology of KGN cells transfected with empty vector (control) or FHL2-expressing vector (FHL2). Cells were cultured in serum-free medium for 21 days before morphological analysis. Scale bar: 200µm. **b**) Viability of KGN cells transfected with empty vector (control) or FHL2-expressing vector (FHL2). Cells were starved (in serum-free medium) for 21 days and stained with the Annexin V-APC/PI dual staining kit before measuring with flow cytometry. All Experiments were repeated three times and the representative images were shown.

Hua *et al*, Fig. S5
