# Supplementary material for: The four and a half LIM domains 2 (FHL2) regulates ovarian granulosa cell tumor progression via controlling AKT1 transcription
Source: Cell Death Dis. 2016 Jul 14;7(7):e2297–. doi: 10.1038/cddis.2016.207 (PMC4973349; doi:10.1038/cddis.2016.207)
Supplement: Supplementary Figure 6 [file cddis2016207x6.pdf]

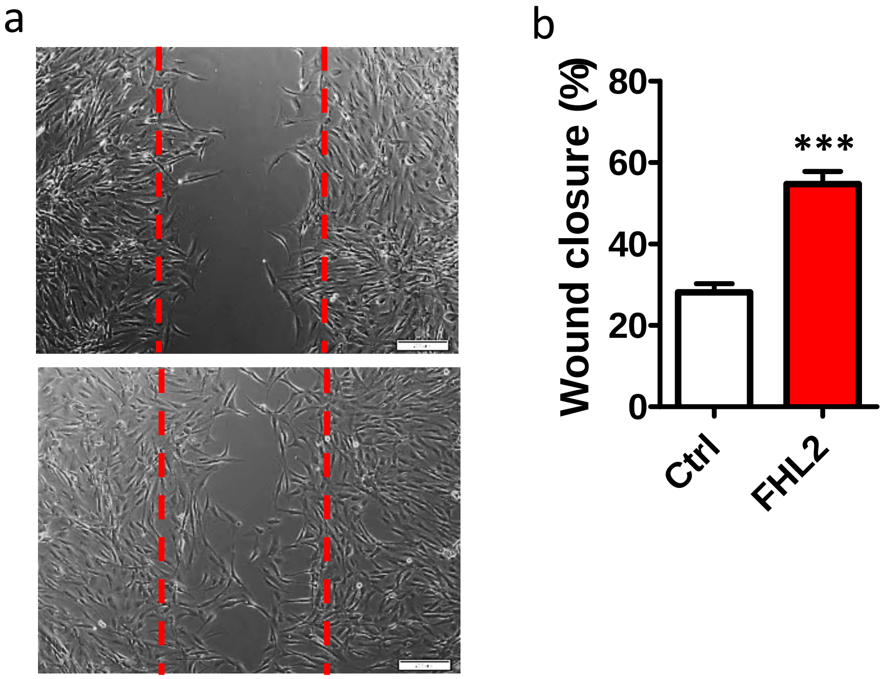

Supplementary figure S6. **Ectopic expression of FHL2 stimulates GCT cell migration. a**) Wound healing assay showing the mobility of KGN cells transfected with empty vector (Ctrl) or FHL2-expressing vector (FHL2). The initial wound edge is highlighted by red dashed lines. cells were incubated for 18h before measuring the wound area. **b**) Quantitative result showing mobility change of KGN cells transfected with empty control vector (Ctrl) or FHL2-expressing vector (FHL2). Each bar represents mean ± SEM of five repeats. ***: $P < 0.001$ compared with control group (Ctrl).

Hua *et al*, Fig. S6
