# Supplementary material for: The four and a half LIM domains 2 (FHL2) regulates ovarian granulosa cell tumor progression via controlling AKT1 transcription
Source: Cell Death Dis. 2016 Jul 14;7(7):e2297–. doi: 10.1038/cddis.2016.207 (PMC4973349; doi:10.1038/cddis.2016.207)
Supplement: Supplementary Figure 7 [file cddis2016207x7.pdf]

Supplementary Information

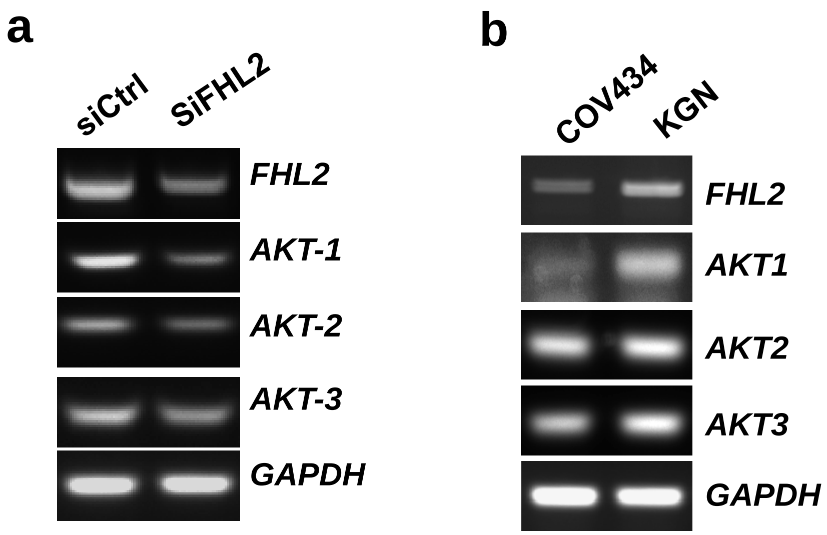

Supplementary figure S7. RT-PCR results showing effects of *FHL2* on *AKT* expression in KGN and COV434 cells. **a**) *FHL2* and *AKT* mRNA levels in KGN cells transfected with non-targeting control siRNA (siCtrl) or FHL2 siRNA (siFHL2). Knockdown of FHL2 suppressed expression of *AKT1*, but not *AKT2* and *AKT3*. **b**) *FHL2* and *AKT* mRNA levels in KGN and COV434 cells. *FHL2* and *AKT* are differentially expressed in FHL2 and COV434 cell lines. Experiments are independently repeated for three times and the representative images are presented.

Hua *et al*, Fig. S7
