# Supplementary material for: The four and a half LIM domains 2 (FHL2) regulates ovarian granulosa cell tumor progression via controlling AKT1 transcription
Source: Cell Death Dis. 2016 Jul 14;7(7):e2297–. doi: 10.1038/cddis.2016.207 (PMC4973349; doi:10.1038/cddis.2016.207)
Supplement: Supplementary Figure 8 [file cddis2016207x8.pdf]

Supplementary Information

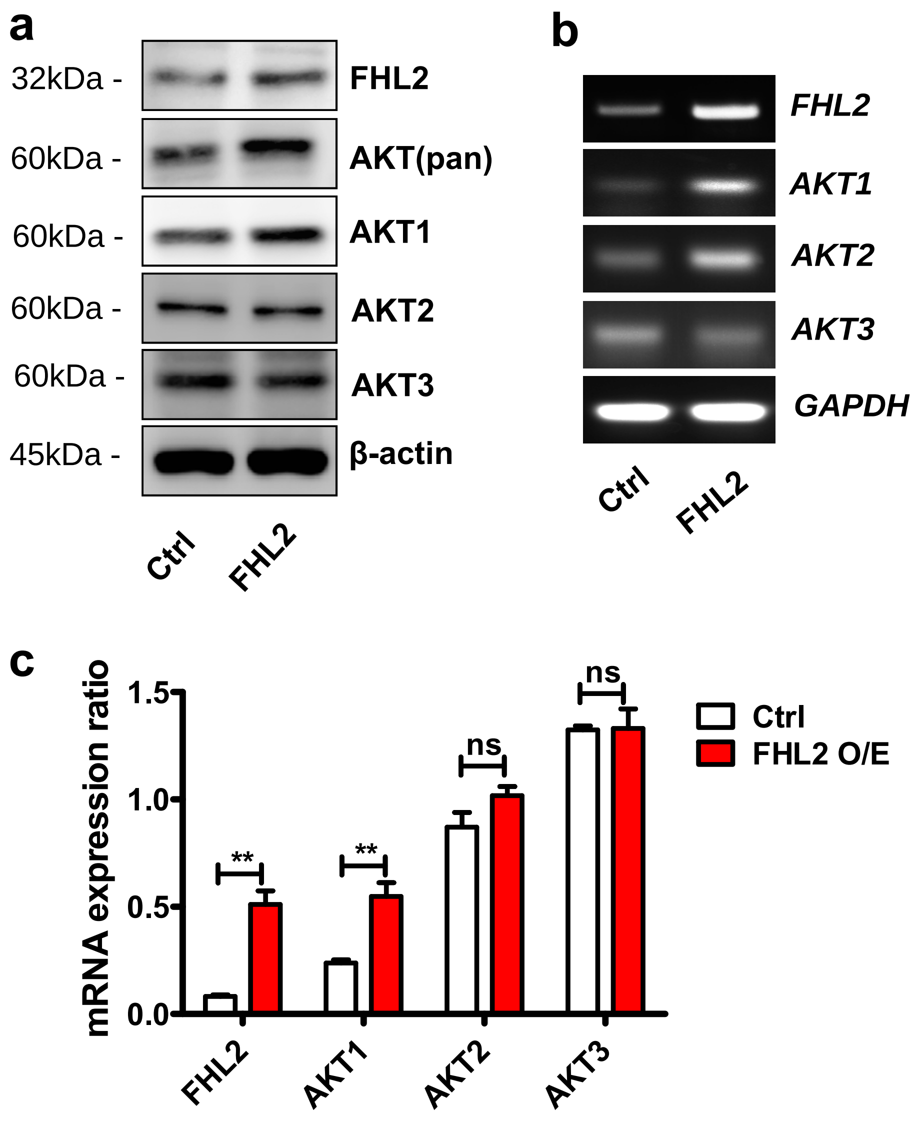

Supplementary figure S8. Overexpression of FHL2 induce AKT1 expression. **a)** Western blot analysis showing that ectopic expression of FHL2 in COV434 cells increased pan-AKT and AKT1 protein levels, but has no effect on protein levels of AKT2 and AKT3. **b**) RT-PCR results showing that ectopic expression of FHL2 in COV434 cells induced increase in the transcription of *AKT1* and *AKT2*, but not *AKT3*. **c**) Quantitative result of figure 5d showing that overexpression of FHL2 induced significant increase in *AKT1*, but not *AKT2* and *AKT3* mRNA in GCT tumor xenografts . Quantitative data were normalized to GAPDH. Bars represents mean ± SEM of four repeats. **: $P < 0.01$ compared to control group (Ctrl). ns: No significant difference.

Hua *et al*, Fig. S8
