# Supplementary material for: The four and a half LIM domains 2 (FHL2) regulates ovarian granulosa cell tumor progression via controlling AKT1 transcription
Source: Cell Death Dis. 2016 Jul 14;7(7):e2297–. doi: 10.1038/cddis.2016.207 (PMC4973349; doi:10.1038/cddis.2016.207)
Supplement: Supplementary Figure 9 [file cddis2016207x9.pdf]

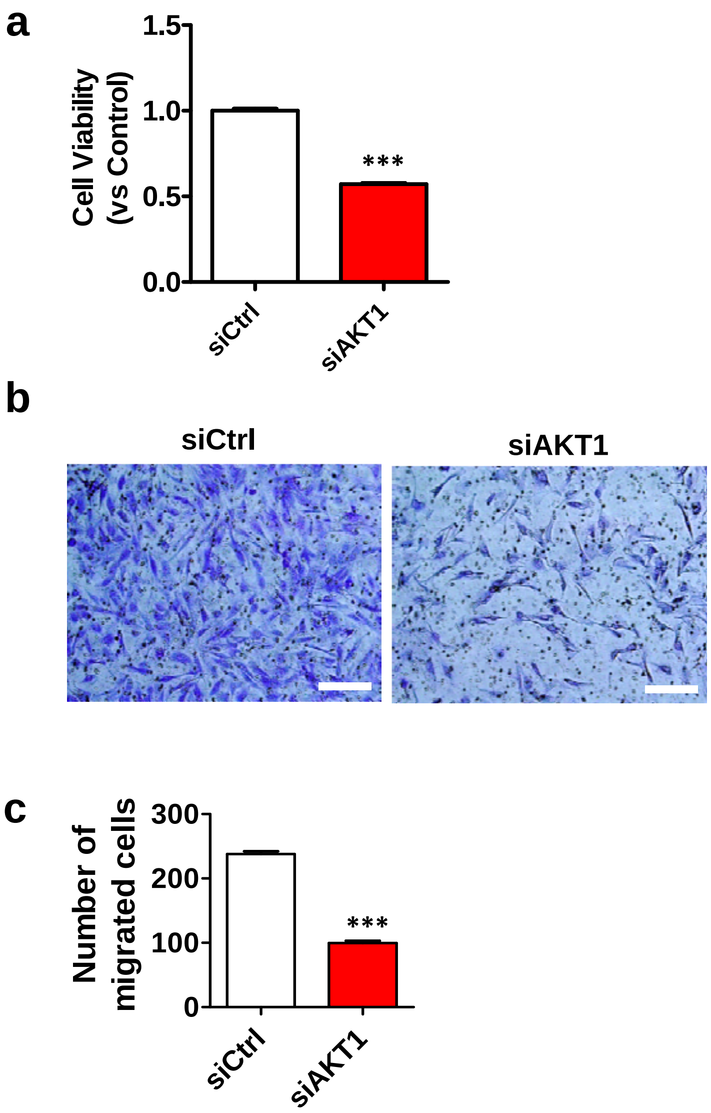


**Supplementary figure S9**. **Knockdown of AKT1 and FHL2 in KGN cells results in similar phenotypes. a**) Change in cell viability in KGN cells transfected with non-targeting control siRNA (siCtrl) or AKT1 siRNA (siAKT1). Cell viability was determined by MTT assay. **b**) Representative images showing migration of KGN cells transfected with non-targeting control siRNA (siCtrl) and AKT1 siRNA (siAKT1). Cell migration was determined using a Transwell migration assay. Scale bar: 200 µm. **c**) Quantitative result of b). Migrated cells were counted manually under a microscope. Each bar represents mean ± SEM (n=5). ***: $P < 0.001$, compared with control (siCtrl).

**Hua *et al*, Fig. S9**
