# Supplementary material for: The four and a half LIM domains 2 (FHL2) regulates ovarian granulosa cell tumor progression via controlling AKT1 transcription
Source: Cell Death Dis. 2016 Jul 14;7(7):e2297–. doi: 10.1038/cddis.2016.207 (PMC4973349; doi:10.1038/cddis.2016.207)
Supplement: Supplementary Figure 10 [file cddis2016207x10.pdf]

Supplementary Information

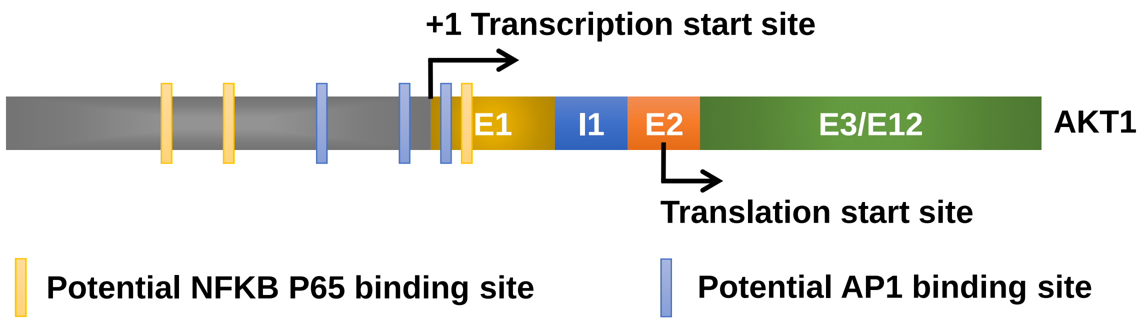

Spplementary figure S10. NFκB and AP-1 transcription factors have two binding sites in *AKT1* gene promoter region. In silico analysis of AKT1 gene with PROMO software (http://alggen.lsi.upc.es/cgi-bin/promo_v3/promo/promoinit.cgi?dirDB=TF_8.3) shows that both NFkB and AP-1 transcription factors have two binding sites in the promoter region of *AKT1* gene. Moreover, both NFκB and AP-1 transcription factors have another binding site in the exon 1 (E1) region of *AKT1* gene..

Hua *et al*, Fig. S10
